# Supplementary material for: Focused low-intensity hippocampal transcranial ultrasound stimulation (TUS) for sleep disturbances in patients with chronic tinnitus: A study protocol for a pilot randomized controlled trial
Source: PLoS One. 2025 Aug 6;20(8):e0329950. doi: 10.1371/journal.pone.0329950 (PMC12327605; doi:10.1371/journal.pone.0329950)
Supplement: S2 File — (PDF) [file pone.0329950.s002.pdf]

Research Protocol

A pilot randomized controlled trial of MRI-guided focused low-intensity transcranial ultrasound stimulation (TUS) for sleep disturbances in patients with chronic tinnitus

Project Title:

A pilot randomized controlled trial of MRI-guided focused low-intensity transcranial ultrasound stimulation (TUS) for sleep disturbances in patients with tinnitus

Study objective (s) and significance

Study Objectives

1) To investigate the safety, feasibility and effectiveness of a 2-week focused low-intensity transcranial ultrasound stimulation (TUS) on the severity of tinnitus and sleep disturbances.
2) To determine the sample size of a full-scale randomized clinical trial of focused low-intensity TUS in patients with chronic tinnitus.
3) To evaluate the effects of focused low-intensity TUS on the severity of tinnitus, sleep quality and cognition at 2, 6 and 12 weeks after the treatments.

Background

Tinnitus is a physical condition, experienced as noises or ringing in a person's ears or head, when no such external physical noise is present. Chronic tinnitus, as a common symptom, could severely jeopardize the sleep quality, brain function, and even lead to hearing loss and cognitive impairments in aging populations (Jafari et al., 2019; Brewster et al., 2022; Loughrey, 2022). Sleep disturbances is a frequent comorbidity in tinnitus sufferers with a prevalence ranging from 50% to 77% (Alster et al., 1993; Hallam, 1996). A growing body of data connects tinnitus to poor sleep quality and an increased risk of cognitive decline (Alster et al., 1993; Jafari et al., 2019). Untreated chronic tinnitus and sleep disturbances can cause frontotemporal brain shrinkage and disruption of default mode network (DMN) and language network as a result of increased listening effort and reduced language function (Chen et al., 2018; De Ridder et al., 2022). The co-occurring chronic tinnitus and sleep disturbances is a slowly progressing preclinical condition that gradually lead to impaired brain function, and reduced functional activities and quality of life with ageing (Nondahl et al., 2007; Pierce et al., 2012).

While the effective management of comorbidities in elderly is important, growing evidence indicates that the disruption of intrinsic neural networks can interfere with the brain activities tagged as DMN, adding to sleep disturbances in tinnitus sufferers (Lan et al., 2020; Milinski et al., 2022; Milinski et al., 2022; Rosemann & Rauschecker, 2023). At present, non-pharmacological therapeutic approaches are very few for elderly patients suffering from chronic tinnitus and sleep disturbances. With an advantage of targeting neural networks, transcranial brain stimulation holds promise for treating chronic tinnitus and sleep disturbances simultaneously. In the last decade, clinical trials of transcranial brain stimulation have shown encouraging results in the treatment of chronic tinnitus. For example, transcranial magnetic stimulation (TMS) over left primary auditory cortex has been reported to improve auditory processing and reduce the perception of tinnitus (Marcondes et al., 2010; Zhang & Ma, 2015; Noh et al., 2020). Although there are a bunch of studies that examine the effects of TMS in treating chronic tinnitus and sleep disturbances, the results are highly varied across clinical trials. The major reasons of this heterogeneity might be summarized into the following three points: (1) Comorbidities: current clinical trial is lacking for TMS that are effective in the management of sleep disturbances in the patients with chronic tinnitus. (2) Discrepancies in treatment targets: beside of primary auditory cortex, left temporoparietal junction (TPJ) and left dorsolateral prefrontal cortex (DLPFC) are also employed as targets in the treatment of tinnitus (Piccirillo et al., 2011; Noh et al., 2020). However, TMS has very limited power to reach deep brain regions that may be related to sleep process, such as hippocampus (Wang et al., 2024). (3) Variability in head size and brain morphometry: the electric fields induced by TMS was severely affected by the morphometric features of treatment target at individual level (Lu et al., 2023). Until now, pre-treatment imaging-informed simulation model has not been used in clinical trials. Compared to TMS, low-intensity TUS, as an advanced modality of transcranial brain stimulation, enables to stimulate the deep brain structures with optimized focality and specific frequency.

Collectively, there is insufficient to support a large, full-scale randomized controlled trial (RCT) that involves investigation of the efficacy and sustainability of novel focused low-intensity TUS in patients with co-occurring chronic tinnitus and sleep disturbances. There is also a lack of clinical data that would allow the estimation of the efficacy of

imaging-guided low-intensity TUS or sample size for the full-scale RCT. Thus, this pilot RCT aims to investigate the safety, feasibility, and efficacy of focused hippocampal low-intensity TUS for sleep disturbances in tinnitus patients that allows to determine the sample size of a full-scale RCT. The findings of this clinical trial will provide valuable clinical evidence that could inform the effect size and personalized modeling of focused low-intensity TUS for age-related brain diseases. Furthermore, the changes of circadian rhythms and auditory function observed in this clinical trial will be helpful for in-depth understanding the relationship of "sleep, hearing and cognition" in late adulthood and guiding the future studies of tinnitus and brain diseases.

Project duration

12 months

Research plan and methodology

Research Design

Randomized, double blind, controlled clinical trial

Methodology

Sources of data

Participants will be recruited through existing research cohort, i.e., Hong Kong cohort of Abnormal Sleep in Ageing Population (HK-ASAP). The detailed medical history of potential participants will be retrieved from the Hospital Authority (HA) system. The research team, including neuroscientist, psychologist and psychiatrists, will identify the participants with chronic tinnitus and sleep disturbances. Eligible participants will be invited to be screened by a trained research assistant to determine the eligibility and availability to participate in the study. Both participants and their caregivers will be briefed about the study before a decision for informed consent.

Potential elderly patients with chronic tinnitus and sleep disturbances will need to satisfy the following inclusion criteria:

1. Chinese, right-handed, aged from 60 to 80 years.

2. Chronic tinnitus is defined as tinnitus with a duration of at least 3 months. Depending on the justification, different time course definitions of chronic tinnitus are possible (De Ridder et al., 2021).

3. Sleep disturbances are defined as a Pittsburgh Sleep Quality Index (PSQI) total score above 5 (Lu et al., 2023).

4. No interference with independence in everyday activities.

Exclusion criteria include:

1. Diseases of ear canal and tympanic membrane checked by otoscopic examination.

2. Previous diagnosis of Meniere's disease and acoustic neuromas.

3. Past history of neurological or mental disorders.

4. Physically frail affecting attendance to treatment sessions.

5. Already attending regular treatments, such as cognitive behavioral therapy or music therapy.

6. Taking a psychotropic or other medication known to affect hearing functions.

7. Significant communicative impairments.

Neuroimaging

All neuroimaging scans will be collected at the Prince of Wales Hospital using a 3.0 Tesla Siemens MAGNETOM Prisma MRI scanner using a 32-channel head coil. High resolution T1-weighted structural magnetic resonance imaging (MRI) scans will be acquired using a Magnetization Prepared RApid Gradient Echo (MPRAGE) sequence with the following parameters: axial acquisition with a 256×256×192 matrix, thickness = 1 mm, no gap, field of view (FOV) = 230 mm, repetition time (TR) = 2070 ms, echo time (TE) = 3.93 ms, flip angle = 15◦. The sequence yields high quality isotropic images with the voxel size of 1 mm ×1 mm × 1 mm. After T1-weighted MRI scanning, diffusion-weighted imaging (DWI) will be acquired in 30 independent directions along with five interleaved non-diffusion weighted (b = 0) images (1.9 mm^3 resolution, TR = 8500 ms, TE = 81 ms, b = 700, echo spacing = 0.69 ms, GRAPPA iPAT factor = 2, 72 slices, 243 ×

243 × 137 mm FOV). An accompanying phase map image was acquired using the same shim as the DWI sequence to correct for field inhomogeneities (4 mm^3 resolution, TR = 1000 ms, TE = 3.60/6.06 ms, FA = 90°, 48 slices, 256 × 256 × 230 mm FOV).

Randomization and masking

Participants will be randomly assigned 1:1 to one of the possible treatments: 1) low-intensity TUS; 2) sham TUS. In order to ensure equally allocation between treatment groups, prior to opening the study for enrollment, the randomization assignment will be generated using an online system (http://randomization.com/) by a statistician not involved in the study design. Assessment staff and participants will be blinded to the study design and group allocation.

Treatment schedule

This pilot RCT is a 2-week treatment with three sessions per week, 80 seconds per session. All participants will receive a total of six sessions of TUS treatment. The schedule for treatment is the same in two randomized groups.

Treatment strategies

Apparatus and settings

NeuroFUS TPO and CTX-500-4 transducer (Brainbox Ltd., Cardiff, UK) are used in this clinical trial. This TUS system consisted of a four-element ultrasound transducer (64 mm diameter) with a central frequency of 500 kHz. We used the theta-burst TUS protocol with the following parameters: pulse duration = 20 ms, pulse repetition interval = 200 ms and total duration = 80 s, giving a total of 400 pulses. The target free field spatial-peak pulse-average intensity (I_{SPPA}) was kept constant at 33.8 W/cm 2 for each participant. We performed transcranial acoustic simulations to ensure that we remained below the FDA guidelines for diagnostic ultrasound (MI ≤ 1.9; $I_{SPPA} \leq 190$ W/cm^2) after transcranial transmission. In addition, we ensured that the maximum temperature rise across the entire 80 s duration of TUS did not exceed 2°C in all our thermal simulations. We prepared each participant's head by parting any hair over the intended target and applying ultrasound transmission gel (Aquasonic 100, Parker Laboratories Inc.). We applied

ultrasound gel to the transducer, used a gel pad (Aquaflex, Parker Laboratories Inc.) and, as far as practically possible, ensured no air bubbles between the transducer face and participant's head.

Stimulation modalities

1. Focused low-intensity transcranial ultrasound stimulation (TUS)

The stimulation parameters of TUS include Pulse duration = 20 ms, pulse repetition interval = 200 ms and total duration = 80 s, giving a total of 400 pulses (Yaakub et al., 2023).

2. Sham TUS

Sham TUS is delivered in the same way as low-intensity TUS, except that the power to the transducer will be turned off. To control auditory effects, we played a sound mimicking the pulse repetition and duration of low-intensity TUS.

Group assignment

All eligible participants will receive a total of six sessions of TUS treatment. According to the modalities of TUS treatment, the participants will be randomly assigned to two groups:

1. Low-intensity TUS (500 kHz)

2. Sham TUS

Randomization

This is a randomized, double-blind, controlled clinical trial. All the participants will be blinded to the grouping information. Independent research assistants who collect the inventory for the severity of tinnitus, sleep quality and cognitive functions will be blinded and will not participate in other outcome assessments.

Outcome assessments

Primary outcomes

1. Subjective sleep quality:

The Pittsburgh Sleep Quality Index (PSQI), as a 19-item self-report questionnaire is used to evaluate the subjective sleep quality in a month (Buysse et al., 1989; Lu et al., 2023). The items produce seven component scores including subjective sleep quality (component 1, C1), sleep onset latency (component 2, C2), total sleep duration (component 3, C3), sleep efficiency (component 4, C4), sleep disturbances (component 5, C5), use of sleep medication (component 6, C6), and daytime dysfunction (component 7, C7). The subscore of each component ranges from 0 to 3, and the maximum total composite score of the PSQI is 21. The sum of these component scores yields a measure of global sleep quality. The cutoff score of poor sleep quality is 5 or more. This Chinese version of the PSQI has been validated with adequate reliability in cognitively intact elderly and dementia patients (Blackwell et al., 2014).

2. Severity of tinnitus

Tinnitus Primary Functions Questionnaire (TPFQ): A short 12-item version of TPFQ is used in this study (Xin et al., 2023). In the 12-item version, questions 7, 11, and 15 were chosen for concentration; questions 4, 8, and 10 were chosen for emotion; questions 2, 14, and 17 were chosen for hearing; and questions 16, 18, and 20 were chosen for sleep.

Secondary outcomes

1. Objective sleep quality: Actigraphic records were used to quantify sleep-wake cycle and estimate the objective sleep efficiency. The actigraph is about the size of a wristwatch and is usually worn on the wrist continuously for multiple days and nights, which can be used as an objective measurement of sleep quality and sleep-wake cycle in older adults.
2. Tinnitus Handicap Inventory (THI) is used to identify, quantify and evaluate the difficulties that patients with chronic tinnitus may experience in daily life. There are 25 items covering three aspects: functional, emotional and catastrophic (Newman et al., 1996). The THI score ranges from 0 to 100, with higher scores indicating more severe tinnitus bur-den and a seven-point change representing a minimal clinically important difference (Gninenko et al., 2024).
3. The Tinnitus Handicap Inventory (THI), as a reliable 25-item self-administered tool, is widely used in the studies of tinnitus treatment efficacy. The THI Chinese version

(THI-CH) has been validated for clinical and research purposes in Hong Kong (Kam et al., 2009).

4. Glymphatic function is assessed by the index of Diffusion Tensor Image Analysis ALong the Perivascular Space (DTI-ALPS) through DWI data (Ma et al., 2024).

Statistical analysis

The data analyst will be blinded to the grouping of participants. Analyses will be on an intention-to-treat basis. Linear mixed models will be used to assess the differences between conditions on the primary and secondary outcome measures at each time point. This statistical method will facilitate inclusion of participants with missing data. Intervention, time points, and their interaction will be modelled as fixed effects. Participants will be modelled as random effects at time points. Pre-intervention tinnitus symptoms, sleep quality and cognitive performance will be compared between the two randomized groups. Score changes of tinnitus symptoms, sleep quality, and cognitive functions from baseline to follow-up points across randomized groups will be tested with occasions (time points) at level one and participants at level two. Covariates identified from baseline differences will be entered in the regression model. Secondary analyses of groupwise differences in the changes of hearing function, memory, and the associations between changes of tinnitus symptoms and sleep quality will be performed. We will also monitor the incidence of adverse events and the characteristics of program adherence. Statistical significance will be set at 2-sided $p < 0.05$. Computations will be performed using *R* Studio (version 1.1.456).

Sample size calculation

As this is a pilot and feasibility study, the sample size is considered seven chronic tinnitus patients with sleep disturbances in each study arm. This sample size is appropriate for the primary and secondary study aims. To evaluate the potential efficacy of the treatments as compared with the control and assuming a medium standardised effect size (0.5), seven participants are required in each group with 80% on-sided CI approach which is suggested for pilot trials (Cocks & Torgerson, 2013). To account for the follow-up rate of 10%, the total sample size is calculated as fourteen.

Ethical considerations

Ethics standards will be strictly followed by providing informed consent and respecting anonymity, privacy and confidentiality. Participants will be recruited if the participants are considered mentally fit to sign consent. No personal identity, including name, birth date, mobile numbers, will be revealed in any reports or publications. Participants can withdraw anytime without interference from any future service use. For those who have any medical concerns during the study, they will be advised to seek help from clinical doctors.

Ethics approval will be obtained from the Clinical Research Ethics Committee (The Joint CUHK-NTEC CREC). This protocol has been registered with the Clinical Trials Registry (NCT06776705). The reporting of trial will follow requirements of major international journals. The study will comply with the Declaration of Helsinki and the Good Clinical Practice (GCP) guidelines of the International Conference on Harmonisation (ICH) of technical requirements for registration of pharmaceuticals for human use (ICH-GCP).

References

Alster, J., Shemesh, Z., Ornan, M., & Attias, J. (1993). Sleep disturbance associated with chronic tinnitus. Biological psychiatry, 34(1-2), 84-90.

Blackwell, T., Yaffe, K., Laffan, A., Ancoli-Israel, S., Redline, S., Ensrud, K. E., & Stone, K. L. (2014). Associations of objectively and subjectively measured sleep quality with subsequent cognitive decline in older community-dwelling men: the MrOS sleep study. Sleep, 37(4), 655-663.

Brewster, K. K., Deal, J. A., Lin, F. R., & Rutherford, B. R. (2022). Considering hearing loss as a modifiable risk factor for dementia. Expert review of neurotherapeutics, 22(9), 805-813.

Buysse, D. J., Reynolds III, C. F., Monk, T. H., Berman, S. R., & Kupfer, D. J. (1989). The Pittsburgh Sleep Quality Index: a new instrument for psychiatric practice and research. Psychiatry research, 28(2), 193-213.

Chen, Y. C., Zhang, H., Kong, Y., Lv, H., Cai, Y., Chen, H., ... & Yin, X. (2018). Alterations of the default mode network and cognitive impairment in patients with unilateral chronic tinnitus. Quantitative imaging in medicine and surgery, 8(10), 1020.

De Ridder, D., Schlee, W., Vanneste, S., Londero, A., Weisz, N., Kleinjung, T., ... & Langguth, B. (2021). Tinnitus and tinnitus disorder: Theoretical and operational definitions (an international multidisciplinary proposal). Progress in brain research, 260, 1-25.

De Ridder, D., Vanneste, S., Song, J. J., & Adhia, D. (2022). Tinnitus and the triple network model: a perspective. Clinical and Experimental Otorhinolaryngology, 15(3), 205-212.

Gninenko, N., Trznadel, S., Daskalou, D., Gramatica, L., Vanoy, J., Voruz, F., & Haller, S. (2024). Functional MRI Neurofeedback Outperforms Cognitive Behavioral Therapy for Reducing Tinnitus Distress: A Prospective Randomized Clinical Trial. Radiology, 310(2), e231143.

Hallam, R. S. (1996). Correlates of sleep disturbance in chronic distressing tinnitus. Scandinavian audiology, 25(4), 263-266.

Jafari, Z., Kolb, B. E., & Mohajerani, M. H. (2019). Age-related hearing loss and tinnitus, dementia risk, and auditory amplification outcomes. Ageing research reviews, 56, 100963.

Lan, L., Li, J., Chen, Y., Chen, W., Li, W., Zhao, F., ... & Cai, Y. (2021). Alterations of brain activity and functional connectivity in transition from acute to chronic tinnitus. Human Brain Mapping, 42(2), 485-494.

Loughrey, D. G. (2022). Is age-related hearing loss a potentially modifiable risk factor for dementia?. The Lancet Healthy Longevity, 3(12), e805-e806.

Lu, H., Chan, S. S. M., Chan, W. C., Lin, C., Cheng, C. P. W., & Linda Chiu Wa, L. (2019). Randomized controlled trial of TDCS on cognition in 201 seniors with mild neurocognitive disorder. Annals of clinical and translational neurology, 6(10), 1938-1948.

Lu, H., Fung, A. W., Chan, S. S., & Lam, L. C. (2016). Disturbance of attention network functions in Chinese healthy older adults: an intra-individual perspective. International Psychogeriatrics, 28(2), 291-301.

Lu, H., Li, J., Yang, N. S., Lam, L. C. W., Ma, S. L., Wing, Y. K., & Zhang, L. (2023). Using gamma-band transcranial alternating current stimulation (tACS) to improve sleep quality and cognition in patients with mild neurocognitive disorders due to Alzheimer’s disease: A study protocol for a randomized controlled trial. Plos one, 18(8), e0289591.

Lu, H., Ma, S. L., Chan, S. S. M., & Lam, L. C. W. (2016). The effects of apolipoprotein ε 4 on aging brain in cognitively normal Chinese elderly: a surface-based morphometry study. International Psychogeriatrics, 28(9), 1503-1511.

Lu, H., Ni, X., Fung, A. W., & Lam, L. C. (2018). Mapping the proxies of memory and learning function in senior adults with high-performing, normal aging and neurocognitive disorders. Journal of Alzheimer's Disease, 64(3), 815-826.

Lu, Y., Li, G., Ferrari, P., Freisling, H., Qiao, Y., Wu, L., & Ke, C. (2022). Associations of handgrip strength with morbidity and all-cause mortality of cardiometabolic multimorbidity. BMC medicine, 20(1), 1-11.

Luik, A. I., Zuurbier, L. A., Hofman, A., Van Someren, E. J., Ikram, M. A., & Tiemeier, H. (2015). Associations of the 24-h activity rhythm and sleep with cognition: a population-based study of middle-aged and elderly persons. Sleep medicine, 16(7), 850-855.

Marcondes, R. A., Sanchez, T. G., Kii, M. A., Ono, C. R., Buchpiguel, C. A., Langguth, B., & Marcolin, M. A. (2010). Repetitive transcranial magnetic stimulation improve tinnitus in normal hearing patients: a double-blind controlled, clinical and neuroimaging outcome study. European Journal of Neurology, 17(1), 38-44.

Milinski, L., Nodal, F. R., Vyazovskiy, V. V., & Bajo, V. M. (2022). Tinnitus: at a crossroad between phantom perception and sleep. Brain Communications, 4(3), fcac089.

Newman, C. W., Jacobson, G. P., & Spitzer, J. B. (1996). Development of the tinnitus handicap inventory. Archives of Otolaryngology–Head & Neck Surgery, 122(2), 143-148.

Noh, T. S., Kyong, J. S., Park, M. K., Lee, J. H., Oh, S. H., Chung, C. K., & Suh, M. W. (2020). Treatment outcome of auditory and frontal dual-site rTMS in tinnitus patients and changes in magnetoencephalographic functional connectivity after rTMS: double-blind randomized controlled trial. Audiology and Neurotology, 24(6), 293-298.

Nondahl, D. M., Cruickshanks, K. J., Dalton, D. S., Klein, B. E., Klein, R., Schubert, C. R., ... & Wiley, T. L. (2007). The impact of tinnitus on quality of life in older adults. Journal of the American Academy of Audiology, 18(03), 257-266.

Piccirillo, J. F., Garcia, K. S., Nicklaus, J., Pierce, K., Burton, H., Vlassenko, A. G., ... & Spitznagel, E. L. (2011). Low-frequency repetitive transcranial magnetic stimulation to the temporoparietal junction for tinnitus. Archives of Otolaryngology–Head & Neck Surgery, 137(3), 221-228.

Pierce, K. J., Kallogjeri, D., Piccirillo, J. F., Garcia, K. S., Nicklaus, J. E., & Burton, H. (2012). Effects of severe bothersome tinnitus on cognitive function measured with standardized tests. Journal of clinical and experimental neuropsychology, 34(2), 126-134.

Rosemann, S., & Rauschecker, J. P. (2023). Disruptions of default mode network and precuneus connectivity associated with cognitive dysfunctions in tinnitus. Scientific Reports, 13(1), 5746.

Sardone, R., Castellana, F., Bortone, I., Lampignano, L., Zupo, R., Lozupone, M., ... & Panza, F. (2021). Association between central and peripheral age-related hearing loss and different frailty phenotypes in an older population in Southern Italy. JAMA Otolaryngology–Head & Neck Surgery, 147(6), 561-571.

Xin, Y., Tyler, R., Yao, Z. M., Zhou, N., Xiong, S., Tao, L. Y., ... & Pan, T. (2023). Tinnitus assessment: Chinese version of the Tinnitus Primary Function Questionnaire. World Journal of Otorhinolaryngology-Head and Neck Surgery, 9(01), 27-34.

Yaakub, S. N., White, T. A., Roberts, J., Martin, E., Verhagen, L., Stagg, C. J., ... & Fouragnan, E. F. (2023). Transcranial focused ultrasound-mediated neurochemical and functional connectivity changes in deep cortical regions in humans. Nature Communications, 14(1), 5318.

Zhang, D., & Ma, Y. (2015). Repetitive transcranial magnetic stimulation improves both hearing function and tinnitus perception in sudden sensorineural hearing loss patients. Scientific Reports, 5(1), 14796.
