# Supplementary material for: Focused low-intensity hippocampal transcranial ultrasound stimulation (TUS) for sleep disturbances in patients with chronic tinnitus: A study protocol for a pilot randomized controlled trial
Source: PLoS One. 2025 Aug 6;20(8):e0329950. doi: 10.1371/journal.pone.0329950 (PMC12327605; doi:10.1371/journal.pone.0329950)
Supplement: Attachment — Submitted filename: Responses to Reviewers comments.pdf [file pone.0329950.s004.pdf]

Point-by-point responses to the reviewer's comments on the manuscript

"Focused low-intensity hippocampal transcranial ultrasound stimulation (TUS) for sleep disturbances in patients with chronic tinnitus: A study protocol for a pilot randomized controlled trial"

Reviewer #1:

Many thanks for the opportunity to review the manuscript titled "Focused low-intensity transcranial ultrasound stimulation (TUS) for sleep disturbances in patients with chronic tinnitus: A study protocol for a pilot randomized controlled trial"

Comment 1: Abstract:

Are you referring to older or senior patients for the target population? What are you referring to as senior patients? Kindly clarify. Be specific on the random assignment procedure, is it 1:1, 1:2 and type of randomization (single, multiple arm etc). What is Sham TUS? Let's use standard nomenclature. Do you mean control? The word sham is a bit problematic.

Response 1:

Thank you so much for reviewing our paper and sharing your valuable suggestions with us. Yes. This study is focusing on older patients with an age range of 60 to 90 years. As you suggested, we have modified the description of sham TUS. Since the word "sham" is a common description of placebo condition in the field of neuromodution, thus, we keep both of the description for clearly defining the placebo condition.

Comment 2: Study Design

Authors have provided details on the study. A two-arm RCT with repeated measurements, including baseline, 2nd week, 6th week and 12th week is a appropriate. However, no justification was provided for implementing the intervention for 12 weeks. Why not 10 or 15 weeks or any other week. Some justification must be provided because the duration of any intervention has significant effect on the outcome measures. Once again, kindly check the use of the word senior. Older adults aged 60-70 years is more appropriate.

Response 2:

Thank you for your insightful comment. All the senior patients have been changed as "older patients". The rationale why we conduct the follow-up assessment at 2nd, 6th and 12th week contains two aspects: 1) The treatment will last for 2 weeks, thus, we need to arrange the 1st time post-treatment assessment within a week. 2) One of our key primary outcomes is subjective sleep quality measured by PSQI, which is a questionnaire that assesses sleep quality and disturbances over a one-month period.

Comment 3: Statistical Methods

The measurement scale (continuous, binary, multinomial, ordinal, etc.) for most primary and secondary outcome measures was unclear. This will inform the kind of statistical models that will be required to quantify the effect of the intervention.

Replace linear mixed models with **linear mixed effect models**. The choice of employing linear mixed Effect models is appropriate, but the measurement scale should be clearly defined to determine whether these models will be appropriate for all the outcome measures. This is important because the authors indicated that the cutoff score for poor sleep quality is 5 or more. If this outcome is treated as binary, then ideally, a mixed-effect logistic regression model will be more appropriate.

Methods to address potential missing observations should be included in the methods section

Response 3: Thank you for your helpful suggestion! The measurement scale has been added in the statistical analysis. For handling missing data, multiple imputation will be used in this study. Considering sleep quality with continuous scale will be used as outcome, we will use linear mixed effect models as you suggested. The modified part in statistical analysis is on Page 13 as "The measurement scales for primary and secondary outcomes are continuous. Linear mixed effect models will be used to evaluate the differences between the conditions on primary and secondary outcomes measured at each time point. This statistical method will facilitate the inclusion of the participants with missing data. For handling missing data, multiple imputation will be used in this study.".

Reviewer #2:

General comment: Thank for the opportunity to review this protocol and pilot randomized control trial. Xi and colleagues propose to investigate the safety and feasibility of applying low-intensity hippocampal TUS for sleep disturbances in chronic tinnitus with the goal of determining a sample size for a future full-scale RCT. The proposed study addresses a clinical gap with the potential for significant impact. The pilot design is appropriate for evaluating feasibility and informing a subsequent full-scale trial. There is a clear recruitment strategy and analysis plan. Overall, my comments are minor and focused on improving the contextualization and rationale for the study, particularly in the introduction. Additionally, the authors may consider clearly defining adverse events and discussing limitations specific to the pilot design explicitly.

Response:

Thank you so much for reviewing our paper and sharing your valuable suggestions with us. A checklist of adverse events associated with low-intensity TUS is applied to monitor tolerability and potential side effects. The Adverse Event Checklist (AEC) covering the symptoms from eight systems, including body as a whole, nervous system, cardiovascular, circulatory, digestive, musculoskeletal, urogenital systems and metabolic, is conducted at each assessment points through this pilot RCT. The updated contents were on page 11.

Comment 1: Introduction

The authors may consider including a brief definition for tinnitus and some common etiologies/ pathophysiology for broader contextualization. Some epidemiology of chronic tinnitus may also help readers to understand the scope of your research question. Additionally, while the authors do establish a relationship between hearing loss and tinnitus, the nature of this association becomes unclear throughout the paragraph. Consider clarifying the interplay between these conditions.

Response 1:

Thank you very much for pointing out this issue. After carefully checking, chronic tinnitus itself does not directly cause hearing loss. Because we do not include a hearing test

(audiogram) Thus, we removed the hearing loss in the contents. Meanwhile, we added a brief introduction in the first paragraph and highlighted the “vicious cycle” of sleep-tinnitus-glymphatic system on Page 4.

Comment 2:

“Growing evidence indicates that the disruption of intrinsic neural networks can interfere with the brain activities tagged as default mode network (DMN), adding to sleep disturbances in tinnitus sufferers” I would suggest providing some more context for this here—why is this important in the context of your study? This is somewhat addressed later on but consider bringing more of that discussion here. Additionally, consider reworking paragraph 2 for improved flow as currently it jumps around quite a bit.

Response 2:

Thank you for this advice. We enhanced this part through introducing the role of DMN in sleep process as “DMN is an intrinsic brain network active during resting state and involved in mind-wandering and memory consolidation. Neuroimaging studies found that sleep disturbances are closely related to the dysfunction of DMN. For example, people with sleep deprivation and insomnia showed weaker hippocampal-DMN connectivity and smaller hippocampal volume. It seems that enhancing sleep quality might be a feasible and indirect pathway for breaking down the vicious cycle and reducing the influence of tinnitus on daily functioning.” on Page 4-5. The newly added references 12-14 have also been updated in the reference list on Page 18.

Comment 3:

“current clinical trial is lacking for TMS that are effective in the management of sleep disturbances and chronic tinnitus simultaneously” Do the authors mean that current TMS trials do not include patients with co-existing tinnitus and sleep disturbance? Or are you citing a specific trial here? Please clarify

Response 3:

Yes. You are right. Previous and current TMS studies focus on single symptom, i.e., either tinnitus or sleep disturbances. In this revision, we listed some results of clinical trials to

clarify the point of view. The newly added references 15-19 have been updated in the reference list on Page 18-19.

Comment 4: Methods-Research Design

Please expand the research design parameters in the text. What measurements will be taken at each timepoint? At which timepoint is TUS delivered? This is nicely summarized in the figure but please include a description in the text as well. Figure 1 is very difficult to read. Text is fuzzy. Consider including a key to define each timepoint.

Response 4:

Thank you for the suggestion. We have clarified the assessment schedule and measurements of assessments as “The comprehensive assessments, including sleep quality, severity of tinnitus, and adverse events, will be conducted at 1 week before low-intensity TUS treatment (baseline, T0), and at 2nd week (T1), 6th week (T2), and 12th week (T3). Actigraphic records and Glymphatic function will be assessed at baseline and 2nd week.” on Page 13. The layout of figure 1 is based on the template of SPIRIT (journal’s template). I am sorry that figure 1 is same as the previous one.

Comment 5: Sample Size and Power Analysis

“To evaluate the potential efficacy of the treatments as compared with the control and assuming a medium standardised effect size (0.5), thirteen participants are required in each group with 80% on-sided CI approach which is suggested for pilot trials.” I believe the authors meant “one-sided.”

Response 5:

Yes. You are right. The typo has been corrected. Thank you!

Comment 6:

Study Population, Recruitment, and Eligibility Criteria

Severe hearing loss is listed as exclusion criteria. How is severe hearing loss defined for your study? Is this based on previous diagnosis, or will patients be screened for hearing loss beforehand? Is there a threshold hearing loss that will not be eligible?

Response 6:

Thank you for your insightful question. We listed severe hearing loss as exclusion criteria based on (1) the medical history and (2) the score of Hearing Handicap Inventory for Adults (HHIA). In the screening stage, we collected the data of HHIA, which is a self-evaluation of hearing loss.

Comment 7:

Ethics: How will your data be handled and stored securely?

Response 7:

The security of clinical trial data is a top priority. In this project, identifiable information, such as ID and age on the MRI images, will be removed entirely to protect participant privacy. The hard copies of the questionnaire will be stored in a locked room. The inputted data will be encrypted during collection and transmission to prevent unauthorized access.

Comment 8:

What qualifies as an adverse event in your study? Who is responsible for monitoring such events and how will they be reported?

Response 8:

A self-reported checklist of adverse events associated with low-intensity TUS is applied to monitor tolerability and potential side effects. The Adverse Event Checklist (AEC) covering the symptoms from eight systems, including body as a whole, nervous system, cardiovascular, circulatory, digestive, musculoskeletal, urogenital systems and metabolic, is conducted at each assessment points through this pilot RCT. The PI (Hanna Lu) and the post-doctoral scientist (Xi Ni) are responsible for monitoring such events.

Comment 9:

Imaging: Clarify within the text at which timepoints in your study design these images (MRI and DWI) will be taken, and for what purpose. In the section on DWI, expand on DTI-ALPS method.

Response 9:

DTI-ALPS is the quantification of the directional diffusion of water molecules with the perivascular space (PVS). The diffusivities in the directions of the x-axis (D_x), y-axis (D_y) and z-axis (D_z) of the regions of interest (ROIs) on projection fibres and association fibres are recorded as D_{xproj}, D_{yproj}, D_{zproj}, D_{xassoc}, D_{yassoc}, D_{zassoc}, respectively.

Comment 10: Simulation Modality

How were these intensity parameters decided upon? Are they based on animal studies?

Response 10:

The intensity parameters were from the recent human studies, such as reference [32] Yaakub SN, White TA, Roberts J, Martin E, Verhagen L, Stagg CJ, Fouragnan EF. Transcranial focused ultrasound-mediated neurochemical and functional connectivity changes in deep cortical regions in humans. Nature Communications. 2023; 14(1): 5318.

Comment 11: Discussion

Consider including the rationale for hippocampal brain region directed TUS. Is this based on previous research in TMS or TUS? Underlying pathophysiology of tinnitus or sleep disturbance?

Response 11:

Thank you for your comment. Yes, the rationale is based on the previous studies of TMS. We have included the contents as "With the advantage of stimulating deep brain structures, low-intensity TUS has great potential for effectively modulating the activities of DMN, particularly hippocampus." on Page 14.

Comment 12: Please include a limitations section.

Response 12:

The limitation section has been updated on **Page 15**. Although pilot RCT is essential for a full-size clinical trial, there are several key limitations that should be carefully considered when interpretating the results. First, pilot RCT involves a small sample size of participants which may reduce the statistical power. Second, the primary aim of pilot RCT

is to test the feasibility of a novel treatment that has underpower to detect small but clinically meaningful effects. Third, this pilot RCT is an offline TUS treatment. Although pre-treatment simulation will be used for locating the target, no online EEG or fMRI will be applied for verifying whether TUS actually modulates the hippocampus. Fourth, objective assessment of hearing function was not included in this study. Fifth, the sample size calculation was based on a medium effect size (0.5) without prior TUS data. Due to the limited sample size, the results may not be generalizable to larger populations. Their limitations highlight the need for cautious interpretation and proper planning for subsequent full-scale clinical trials.

Reviewer #3:

General comment: The manuscript is well-structured and presents a compelling study protocol for a pilot RCT investigating low-intensity transcranial ultrasound stimulation (TUS) for sleep disturbances in chronic tinnitus patients. Below is a detailed review with suggestions for improvement.

Suggestion 1: Abstract:

(1) Specify the primary/secondary outcomes more succinctly (e.g., "Primary: PSQI; Secondary: actigraphy, THI, DTI-ALPS"); (2) Clarify the intervention duration (e.g., "2-week treatment, 3 sessions/week, 80s/session").

Response 1:

Thank you so much for reviewing our paper and sharing your valuable suggestions with us! As you suggested, the outcome measurements have been added in the abstract on Page 3.

Suggestion 2: Introduction: Streamline the "vicious cycle" explanation (glymphatic dysfunction → tinnitus → sleep disruption) to avoid redundancy.

Response 2:

Thank you for your suggestion. We have deleted some words for avoiding redundancy. However, we believe that a very short description of glymphatic function is necessary for explaining the "vicious cycle". Thus, we kept this part in the main contents.

Suggestion 3: Define DMN (Default Mode Network)

Response 3:

The definition and role of DMN has been updated on Page 4. DMN is an intrinsic brain network active during resting state and involved in mind-wandering and memory consolidation. Neuroimaging studies found that sleep disturbances are closely related to the dysfunction of DMN. For example, people with sleep deprivation and insomnia showed weaker hippocampal-DMN connectivity and smaller hippocampal volume. It seems that enhancing sleep quality might be a feasible and indirect pathway for breaking down the vicious cycle and reducing the influence of tinnitus on daily functioning.

Suggestion 4:

Clear justification for TUS over TMS (e.g., deeper targeting of hippocampus, glymphatic modulation).

Response 4:

Yes. The contents have been modified on page 5, as “Compared to TMS, focused low-intensity transcranial ultrasound stimulation (TUS) is a newly developed modality of NIBS that offers promising therapeutic effects by modulating the glymphatic system and transmitting acoustic energy into brain parenchyma. Low-intensity TUS can reach deeper brain regions, such as hippocampus, with a high spatial resolution (i.e., mm), which sparks interest in targeting DMN and managing the comorbidities in ageing populations.”

Suggestion 5: Methods:

Randomization: Clarify how blinding is maintained (e.g., who administers TUS vs. assesses outcomes?). Sham Protocol: Reiterate how auditory masking ensures blinding (currently mentioned but could be emphasized).

Response 5:

Assessment staff and participants will be blinded to the study design and group allocation. The researcher who administers the TUS treatment will not participate in the assessments. The sham TUS protocol was aligned with the reference: Yaakub, S. N., Bault, N., Lojkiewiez, M., Bellec, E., Roberts, J., Philip, N. S., & Fouragnan, E. F. (2024). Non-invasive Ultrasound Deep Neuromodulation of the Human Nucleus Accumbens Increases Win-Stay Behaviour. bioRxiv, 2024-07.

Suggestion 6:

Glymphatic Measurement: Briefly explain DTI-ALPS and its validation in tinnitus/sleep studies (or cite references). How will you verify TUS actually modulates the hippocampus? (fMRI/EEG planned?)

Response 6:

Thank you for pointing out this issue. We fully acknowledge the importance of online settings of TUS. Unfortunately, neither EEG nor fMRI will be included in this pilot clinical

trial. Thus, we mentioned this part in limitations as “Third, this pilot RCT is an offline TUS treatment. Although pre-treatment simulation will be used for locating the target, no online EEG or fMRI will be applied for verifying whether TUS actually modulates the hippocampus.” on Page 15.

Suggestion 7:

Why choose a 2-week intervention? Is this based on prior TUS durability data?

Response 7:

Yes. Another consideration is safety.

Suggestion 8:

How will you handle missing data (e.g., multiple imputation)?

Response 8:

Yes. We understand the importance of handling missing in a clinical trial. In this study, three times of follow-up assessments are included. For enhancing the engagement of participants, kind reminders and incentives will be applied. For better handling missing data, multiple imputation will be used to create multiple plausible imputed datasets, analyze each separately, and then combine the results.

Suggestion 9: Statistical Analysis

Power Analysis: Acknowledge limitations of assuming a medium effect size (0.5) without prior TUS data. Covariates: List potential covariates (e.g., baseline PSQI, tinnitus duration, age) to be included in mixed models.

Response 9:

Thanks a lot for your suggestion. In this revision, the limitation part has been modified as “Fifth, the sample size calculation was based on a medium effect size (0.5) without prior TUS data. Due to the limited sample size, the results may not be generalizable to larger populations.” on Page 15. The statistical analysis has been modified as “Covariates, including age, duration of tinnitus and PSQI total score, identified from baseline differences will be entered in the regression model.” on Page 13-14.
