# Supplementary material for: Focused low-intensity hippocampal transcranial ultrasound stimulation (TUS) for sleep disturbances in patients with chronic tinnitus: A study protocol for a pilot randomized controlled trial
Source: PLoS One. 2025 Aug 6;20(8):e0329950. doi: 10.1371/journal.pone.0329950 (PMC12327605; doi:10.1371/journal.pone.0329950)
Supplement: Attachment — Submitted filename: Responses to Reviewers comments.docx [file pone.0329950.s005.docx]

**Point-by-point responses to the reviewer’s comments on the manuscript**

“Focused low-intensity hippocampal transcranial ultrasound stimulation (TUS) for sleep disturbances in patients with chronic tinnitus: A study protocol for a pilot randomized controlled trial”

**Reviewer #1:**

Authors have addressed all my previous comments and also provided clarity when the need arises. The manuscript has improved significantly.

**Response 1:**

Thanks again for reviewing our paper and sharing your valuable suggestions with us.

**Reviewer #2:**

**General Comment:**

Thank you for the opportunity to review the revised version of this manuscript. The authors have addressed many of the concerns raised in the initial review, and the paper has improved in clarity and rigor. Below I outline a few remaining issues and suggestions for further strengthening the manuscript.

**Response:**

Thank you for reviewing our paper again and sharing your insightful comments for improving the quality and readability of this protocol paper.

**Comment 1:** Introduction

The authors have effectively revised the introduction to include more background details and epidemiology on tinnitus. However, various sentences in the discussion of the “vicious cycle” remain disjointed and unclear. For example, “Untreated sleep disturbances can cause glymphatic dysfunction and brain atrophy as a result of accelerated cognitive decline in chronic tinnitus sufferers”. The structure of this sentence makes the cause effect relationship unclear implying that cognitive decline causes glymphatic dysfunction and brain atrophy rather than sleep disturbance—> glymphatic dysfunction-> brain atrophy-> cognitive decline. Please rework this section to clarify. The addition of more details about the DMN is effective and helps to clarify the study aims.

**Response 1:**

Thank you very much for pointing out this issue. After carefully checking, this part has been revised as “Of note, glymphatic dysfunction can lead to the accumulation of neurotoxic waste products in the brain, potentially contributing to neurological conditions, such as tinnitus and cognitive decline. Poor sleep quality may exacerbate this dysfunction, creating a vicious cycle in which chronic tinnitus disrupt sleep, and sleep disturbances further compromise glymphatic clearance. Thus, targeting this cycle through interventions that address both sleep disturbances and chronic tinnitus could hold significant therapeutic value, particularly in ageing populations.” On Page 4.**Comment 2:**

“Comorbidities: current clinical trials are lacking for the evidence of using TMS to manage sleep disturbances and chronic tinnitus simultaneously.” While the authors have added the related studies to the references, this sentence remains unclear. Please reword to clarify that current trials focus exclusively on one symptom, either tinnitus or sleep disturbance.

**Response 2:**

Thank you for this advice. This sentence has been re-written as “Comorbidities: current clinical trials only focus on one symptom (i.e., either tinnitus or sleep disturbances) and provide insufficient evidence to support the use of TMS for managing both sleep disturbances and chronic tinnitus simultaneously.” on Page 5.

**Comment 3:** Methods

Please include your justification and parameters for excluding severe hearing loss in the text. Please include details about data handling and security in the ethics section of the text. The authors have included a reference to justify their intensity parameters; however, a brief contextual explanation of this reference is lacking in the text. Please elaborate on how this reference supports your point. The addition of a subheading for safety and adverse events and the subsequent discussion of the adverse events checklist in the methods section is appreciated.

**Response 3:**

Thank you so much for pointing out the issues. In this study, we will check the medical records of the participants. Thus, if the participants are deaf or have severe hearing loss, they will be excluded from this study after screening. We have explained this issue on Page 8.

The data handling and security has been updated on Page 9 as “All participant data, including audiometric results and health surveys, were anonymized at collection and stored on password-protected computers. Access was limited to study investigators, and data retention follows IRB guidelines (5 years post-study).”

Yes. As you mentioned last time, we used the theta-burst TUS protocol in this trial. On Page 8, this part has been rewritten as “Theta-burst TUS (tbTUS) protocol will be used in this study for modulating neuronal activities and enhancing the neuroplasticity in hippocampal circuits [32,33]. The stimulation parameters of low-intensity TUS include Frequency: 5 Hz, pulse duration = 20 milliseconds, pulse repetition interval = 200 milliseconds and the total duration = 80 seconds, giving a total of 400 pulses.”. The reference list has been updated as well.

The subheading has been updated on Page 11.

**Comment 4:** Discussion

Enhanced discussion of targeting deeper brain structures with TUS is effective. Thank you for including a comprehensive limitations section. The authors now acknowledge limitations more clearly, which is appreciated.

**Response 4:**

Thank you for your feedback and comment. We have xxxx.

**Comment 5:**

The following are some language points that can be improved.

Till: use until (abstract)

“Toward effectively managing the co-occurring sleep disturbances and chronic tinnitus, it is fundamental for understanding the neural underpinnings of the vicious cycle” (intro)Suggest rephrasing for clarity (i.e. Understanding the neural underpinnings of the vicious cycle is fundamental to effectively managing co-occuring sleep disturbances and chronic tinnitus.)

“There are a bunch of studies” (intro) This phrase is too informal for scientific writing; consider replacing with “numerous studies have shown…”

Until to date: Revise to “To date,” or “until the present,” or “until now” (methods)

**Response 5:**

Thank you for carefully reviewing our paper. The points mentioned in the comment have been revised on Page 2, Page 4, Page 5 and Page 6.

**Reviewer #3:**

Comments have been addressed and answered completely in prefect way. The manuscript is well-structured and methodology well done No further comments.

**Response:**

Thanks again for reviewing our paper and sharing your valuable suggestions with us.
